# Epigallocatechin-3-gallate inhibits doxorubicin-induced inflammation on human ovarian tissue

**DOI:** 10.1042/BSR20181424

**Published:** 2019-05-14

**Authors:** R Fabbri, M Macciocca, R Vicenti, G Caprara, MP Piccinni, R Paradisi, P Terzano, A Papi, R Seracchioli

**Affiliations:** 1 Gynecology and Physiopathology of Human Reproduction Unit, Department of Medical and Surgical Sciences, University of Bologna, S. Orsola-Malpighi Hospital of Bologna, Italy; 2 Histopathological and Molecular Diagnostic Unit of Solid Organ and Transplant, S. Orsola-Malpighi Hospital, Bologna, Italy; 3 Department of Experimental and Clinical Medicine, Center of Excellence DENOTHE, University of Florence, Florence, Italy; 4 Department of Biological, Geological, & Environmental Science, University of Bologna, Bologna, Italy

**Keywords:** doxorubicin, epigallocatechin-3-gallate, human ovarian tissue, inflammation

## Abstract

Chemotherapy protocol can destroy the reproductive potential of young cancer patients. Doxorubicin (DOX) is a potent anthracycline commonly used in the treatment of numerous malignancies. The purpose of the study was to evaluate the ovarian toxicity of DOX via inflammation and the possible protective effect of the green tea polyphenol epigallocatechin-3-gallate (EGCG). Ovarian tissue of three patients was cultured with 1 µg/ml DOX and/or 10 µg/ml EGCG for 24 and 48 h. Levels of inflammatory factors were determined by quantitative Real-Time PCR, western blot, zimography, and multiplex bead-based immunoassay. Morphological evaluation, damaged follicle count and TUNEL assay were also performed. DOX influenced inflammatory responses by inducing a significant increase in the expression of pro-inflammatory cytokines, such as tumor necrosis factor-α (TNF-α) and cyclooxigenase-2 (COX-2), of inflammatory interleukins (IL), such as interleukin-6 (IL-6) and interleukin-8 (IL-8), and the inflammatory proteins mediators metalloproteinase-2 and metalloproteinase-9 (MMP2 and MMP9). IL-8 secretion in the culture supernatants and MMP9 activity also significantly raised after DOX treatment. Moreover, a histological evaluation of the ovarian tissue showed morphological damage to follicles and stroma after DOX exposure. EGCG significantly reduced DOX-induced inflammatory responses and improved the preservation of follicles. DOX-induced inflammation could be responsible for the ovarian function impairment of chemotherapy. EGCG could have a protective role in reducing DOX-mediated inflammatory responses in human ovarian tissue.

## Introduction

Recent advances in cancer diagnosis and the use of new chemo/radiotherapy protocols have significantly increased survival rates for children and adults with cancer. However, these treatments are gonadotoxic and can severely compromise or totally destroy the reproductive potential of women by inducing premature ovarian failure (POF) [1,2]. The potential iatrogenic loss of fertility has a profound impact on young women and may be more stressful than the cancer diagnosis itself [3]. Doxorubicin (DOX) is an anthracycline commonly used in the treatment of numerous malignancies, such as breast cancer, lymphoma, leukemia, and solid tumors [4–7]. The widespread use of DOX in the young female population requires strategies to counteract undesirable ovarian toxicity. Defining chemotherapy insult on the ovary is complex because of the heterogeneous nature of the gonad, which is composed of stromal cells, follicles, and vessels.

In mice DOX-induced ovarian toxicity is associated with apoptosis of granulosa cells, reactive oxidative specie accumulation and decline of mitochondrial membrane potential in granulosa cells [8,9]. DOX treatment results in a significant loss of ovarian reserve within mice ovaries, mostly secondary follicles, and vascular changes such as thickening and hyalinization of the vessel wall [10]. Moreover, a specific accumulation pattern of DOX is observed in the mice ovaries [11]: the first target of the drug seems to be the stromal compartment, followed by granulosa cells that finally result in follicular apoptosis.

In human ovary DOX causes dsDNA-breaks in primordial follicles, oocytes, granulosa cells, and vascular and stromal damage [12]. One of the causes of DOX-induced damage is the induction of inflammation [13,14] and the overproduction of free radicals [15–17] which cause DNA fragmentation, apoptosis [18–20], and lipid peroxidation [21,22]. It is reasonable to assume that the administration of antioxidant agents concurrently with the chemotherapeutic drug may have a protective effect on the ovary [23–26].

Epigallocatechin-3-gallate (EGCG), the most abundant catechin in green tea, seems to prevent oxidation of low-density lipoprotein. Thanks to its high antioxidant activity, it can protect the cells and DNA from damage [27,28]. Previous studies have confirmed that EGCG has a wide range of pharmacological effects, and has obvious preventive and therapeutic effects as an anti-inflammatory [29], antivirus [30], anticancer [31], and on ulcerative colitis [32], and autoimmune diseases [33]. The antioxidant activity of EGCG is 30 times that of vitamin C and vitamin E [34]. The use of a water-soluble natural antioxidant, commonly used as a dietary supplement, might have the advantage of being easily administrable and ethically acceptable.

The present study aims to investigate the ovarian toxicity of DOX via inflammation and the possible protective effect of EGCG in human ovarian tissue model.

## Materials and methods

### Collection of human ovarian tissue

The Ethics Committee of S. Orsola-Malpighi Hospital of Bologna (74/2001/O) approved the study on human ovarian tissue. Ovarian tissues were obtained from three patients suffering from non-gynecological diseases (mean age ± S.D.: 28 ± 2 years) undergoing ovarian tissue cryopreservation to preserve their ovarian function before receiving anticancer therapies. All women donated their tissue for research purpose.

The ovarian biopsy was retrieved by laparoscopy and immediately transferred to the laboratory in phosphate buffered saline (PBS) medium (Stemcell Technologies, Milan, Italy) supplemented with 10% human serum (provided by the Transfusion Center of S.Orsola-Malpighi Hospital, Bologna, Italy) at 4°C. The medulla of the biopsy was removed using a surgical scissor, and the cortical tissue was dissected in strips (±1 cm × 2 mm × 1 mm), and slowly frozen according to the protocol previously described [35].

### Ovarian tissue culture

For each patient, cortical strips were thawed using a slightly modified protocol described by Fabbri et al. [35] (removal of cryoprotectants at 4°C). After thawing, the cortical strips were cut into 24 small fragments (2 mm × 2 mm) and incubated for 1 h at 37°C and 6% CO_2_ in a basal culture medium (BCM) composed of α minimum essential medium (Sigma, Milan, Italy), 1% insulin–transferrin–sodium selenite (5 μg/ml insulin, 5 μg/ml transferrin, and 5 ng/ml sodium selenite, Sigma), 40% human serum, 25 mmol/l N-acetyl-cysteine (Sigma), 0.02 µg/ml IGF-II (Sigma) and antibiotics, to allow tissue rebalance before treatments. Subsequently, the ovarian fragments were divided randomly as follows: 12 fragments for each time (24 and 48 h). Then, within each time, three fragments were assigned to control group (CTR), three fragments to DOX group, three fragments to EGCG group, and three fragments to DOX+EGCG group. The fragments were destined to quantitative real-time PCR (qRT-PCR) (one fragment), western blot (one fragment), and histology/immunohistochemistry/TUNEL assay (one fragment).

The fragments were cultured in sterile 1.9 cm^2^ petri dishes (Nunclon™ Delta, Roskilde, Denmark) at 37°C and 6% CO_2_ in 1 ml of: (1) only BCM for CTR, (2) BCM supplemented with 1 µg/ml DOX (Sigma); (3) BCM supplemented with 10 µg/ml EGCG (Sigma), and (4) BCM supplemented with combined treatments 1 µg/ml DOX+10 µg/ml EGCG.

For each experimental condition, culture media were collected for zymography after 24 h of culture and for multiplex bead-based immunoassay after 24 and 48 h of culture.

The concentration of DOX was selected according to previous studies reproducing the peak plasma concentrations reached by standard infusions in patients [36,37] and according to our previous study on human ovarian stromal cells [38]. The concentration of EGCG that was used corresponds to the mean peak plasma levels after drinking the equivalent of approximately two cups of green tea [39] and it was selected according to our previous study on normal human peripheral blood lymphocytes [40]. These cells are very sensitive to toxic molecules and chemicals, and they have often served as a model of clinical toxicity of drugs. The EGCG concentration, that was used in the present study, did not have significant cytotoxic effects on normal human lymphocytes [40].

### RNA extraction and reverse transcription

Total RNA was extracted from tissue after 24 and 48 h of culture by using RNAxpress (Thermo Scientific, Waltham, MA, U.S.A.) reagent after homogenization with a Turex instrument and was quantitated spectrophotometrically. The RNA (1.5 µg) of each sample was reverse transcribed using Revertaid First Strand cDNA Synthesis Kit (Thermo Scientific) following the manufacturer’s protocol.

### Quantitative real-time PCR

RT-PCR analysis of cDNA was performed using a fluorescent nucleic acid dye similar to SYBRGreen (SsoFast EvaGreen Supermix, Bio-Rad. HerculeS, CA, U.S.A.).

We used the 2^-ΔΔCt^ method for relative quantification of gene expression. The final results were determined as follows: N target = 2^−(ΔCt sample − ΔCt calibrator)^, where ΔCt values of the sample and calibrator were determined by subtracting the Ct value of the endogenous control gene from the Ct value of each target gene [41]. The results, presented as fold change (2^-ΔΔCt^), were multiplied for 100. The relative amount (%) of the target genes was calculated using the expression of human actin as an endogenous control. The primer sequences are reported in the Supplementary Table S1. All samples were run in triplicate in 20 μl reaction volume containing 200 ng of cDNA. The thermal cycler was programmed as follows: 30 s at 95°C and 40 cycles of 30 s at 95°C, and 60 s at 60°C for amplification.

### Western blot

Proteins obtained from lysate tissue after 24 and 48 h were size fractioned in 4–12% SDS-polyacrylamide gel, before being transferred to PVDF membrane (GE Healthcare, U.K.) by using Trans-Blot Turbo System (Bio-Rad, U.S.A.). Membranes were blocked for 2 h with 5% bovine serum albumine (BSA) in transfer buffer saline (TBS: 2.5% Tris-HCl, 8% NaCl, 0.1% Tween 20, pH 7.4) at room temperature. Afterward the membranes were incubated in 3% TBS–BSA with primary antibodies overnight at 4°C (Supplementary Table S2).

After two washes in PBS 1×, the membranes were incubated with the secondary conjugated antibodies antimouse Cy3 or antirabbit Cy5 (GE, U.S.A.) diluted 1:2500 in 3% TBS-BSA for 2 h (Supplementary Table S2). Immunolabeling was visualized using the ECL Plex procedure (GE) and the laser scanner Pharos FX (Bio-Rad). Bands were quantitated using densitometric image analysis software (Bio-Rad) or ImageJ software. Molecular mass was determined using a wide range protein marker 8–200 kDa (Bio-Rad). Protein loading was controlled by antiactin (Sigma) detection and was statistically evaluated.

### Zimography

MMP2 and MMP9 activity were measured in 24 h culture media by zimography assay. After centrifugation (300 *** g*** for 10 min), the supernatant was separated and the protein concentrations were measured using Bradford protein assay. Total 20 µg of proteins per lane were added to the sample buffer (1 M Tris-HCl, pH 6.8, 2% sodium dodecyl sulphate (SDS), and 10% glycerol] and loaded onto a 10% SDS–polyacrylamide gel containing 1 mg/ml gelatine porcine (Sigma). Gel was stained in 0.1% Coomassie Brilliant Blue R-250 for approximately 1 h and destained until the gelatino-lytic bands were clearly seen. The MMP activities, indicated by clear bands of gelatin digestion on a blue background, were quantitated using densitometric image analysis software (Bio-Rad).

### Multiplex bead-based immunoassay

The concentrations of TNF-α, IL-6, and IL-8 were measured in 24 and 48 h culture media using the xMAP technology (bead-based multiplex immunoassay). Multiplex bead-based immunoassay allows to measure multiple analytes simultaneously in individual samples by flow cytometric resolution of spectrally distinct microspheres paired with monoclonal antibodies specific for cytokines and reporter fluorochromes bound to detection antibodies. The bead-based multiplex sandwich immunoassay (Bio-Rad) was read with a Luminex system (Luminex Map Technology, Milan, Italy) In brief, 50 µl of each individual culture medium were added to 50 µl of antibody-conjugated beads directed against the cytokines (Bio-Rad) in a 96-well filter plate (Bio-Rad). After a 30-min incubation, the plate was washed and 25 µl of biotinylated anti-cytokine antibody solution were added to each well before another 30 min incubation. The plate was then washed and 50 µl of streptavidin-conjugated PE were added to each well. After a final wash, each well was resuspended with 125 µl of assay buffer (Bio-Rad) and analyzed using the Luminex array system.

### Histology – immunohistochemistry – TUNEL assay

Tissue fragments were fixed after 24 and 48 h of culture in 4% formaldehyde, embedded in paraffin blocks, and cut in 4 μm thick sections. Every 50 μm, one section was collected: the first, the third, and the fifth sections were stained with hematoxylin and eosin (Merck, Darmstadt, Germany); the second, the fourth, and the sixth sections were used for immunohistochemistry and the seventh, the eighth and the ninth sections were collected for terminal deoxynucleotidyl transferase dUTP nick end labeling (TUNEL) analysis. The histological sections were used to assess follicle count, developmental follicle stage according to Gougeon classification [42], follicle, and stroma preservation. Follicles having oocyte nuclei with dispersed chromatin, homogeneous cytoplasm with perinuclear localization of mitochondria aggregates and no vacuolization, and granulosa cells allocated tightly around the oocyte, were considered preserved. Stroma was considered preserved if the cells had oval shaped nuclei with finely dispersed chromatin and no cytoplasm vacuolization, and if no interstitial oedema was present.

For immunohistochemistry, the sections were incubated overnight at 4°C with the following primary antibodies: anti-Ki-67 (1:80, Bio Genex, San Ramon, CA, U.S.A.) and anti-Bcl2 (1:80, Dako, Carpinteria, CA, U.S.A.). An En Vision monoclonal immunoenzymatic system was used for immunnohistochemical detection (Dako). The reaction was developed in 3,3-diaminobenzidine (DAB, Sigma, St. Louis, MO, U.S.A.). Finally, the sections were counterstained with Mayer’s hematoxylin for 10 s, dehydrated, and mounted in Eukitt. Control procedures were undertaken simultaneously to ensure the specificity of immunostaining [43]. Sections without primary antibodies were used as a negative control (Supplementary Figure S1), and sections of human breast cancer were used as a positive control. Follicle and stromal positivity for the primary antibodies were evaluated at 200× magnification under a Leitz microscope. Follicles were considered positive if the nucleus of the oocyte and/or at least one nucleus of the granulosa cells resulted stained.

For TUNEL analysis, the cell death detection kit conjugated with horseradish peroxidase (Roche, Mannheim, Germany) was used according to the manufacturer’s instructions. As positive control, sections were incubated with DNase I (3000 U/ml in 50 mM Tris–HCl, pH 7.5, 1 mg/ml BSA) for 10 min at 15–25°C to induce DNA strand breaks (Supplementary Figure S2), prior to labeling procedure; as negative control, sections were incubated with label solution only (without terminal transferase) instead of TUNEL reaction mixture. The apoptotic signal was recorded as positive when dUTP stained the nucleus brown. Follicles were considered damaged when the oocyte nucleus and/or more than two of granulosa cells were stained in brown by dUTP. The number of apoptotic stromal cells was analyzed in 100 mm^2^ (randomly assigned in the sections), counted three times, and then averaged. The percentage of apoptotic cells (apoptotic cell number/total cell number × 100) was quantitated in a double blind fashion using a Leitz Diaplan light microscope equipped with a CCD JVC video camera. Digitized images were analyzed with Image ProPlus software.

### Statistical analysis

All experiments were performed in triplicate. Statistical analysis was assessed by ANOVA followed by Bonferroni’s multiple comparison, using PRISM 5.1 (Graph Pad Software, La Jolla, CA, U.S.A.). Data were expressed as mean ± S.D. *P* value <0.05 was considered statistically significant.

## Results

### Effects of DOX and EGCG on cytokine and IL expression in the human ovarian tissue

We investigated the inflammatory effects of DOX on human ovarian tissue, testing the mRNA expression levels of pro-inflammatory cytokines, such as COX-2 and TNF-α, and inflammatory ILs such as IL-6 and IL-8.

**Figure 1:**
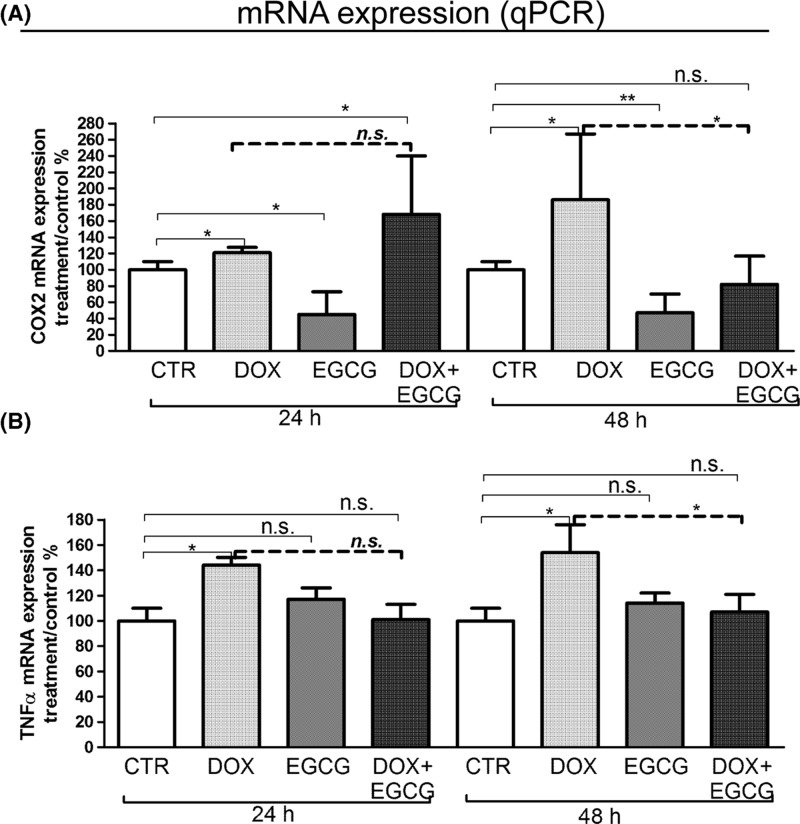
Effects of DOX and EGCG on the ovarian tissue inflammation qRT-PCR for COX-2 and TNF-α. RNA was isolated after 24 and 48 h treatment with DOX 1 μg/ml, EGCG 10 μg/ml, and combined treatment with DOX+EGCG at the aforementioned concentrations. COX-2 (**A**) and TNF-α (**B**) mRNA expression was expressed as a percentage of treated on control samples (CTR). Each bar represents the mean (± S.D.) of three independent experiments. *P<0.05, **P<0.01, n.s.: not significant.

DOX induced a significant increase of COX-2 mRNA expression levels with respect to CTR after 24 and 48 h (*P*<0.05) of treatment. On the contrary, EGCG produced a significant decrease of COX-2 mRNA expression levels when compared with CTR in a time-dependent manner (24 h *P*<0.05; 48 h *P*<0.01). The supplementation of EGCG did not affect DOX-induced COX-2 mRNA expression after 24 h but significantly reduced it after 48 h (*P*<0.05) (Figure 1A).

TNF-α expression significantly increased with respect to CTR after 24 and 48 h of DOX treatment (*P*<0.05). EGCG did not have any effect when used alone but significantly reduced DOX-induced TNF-α mRNA expression after 48 h of combined treatment (*P*<0.05) (Figure 1B).

**Figure 2:**
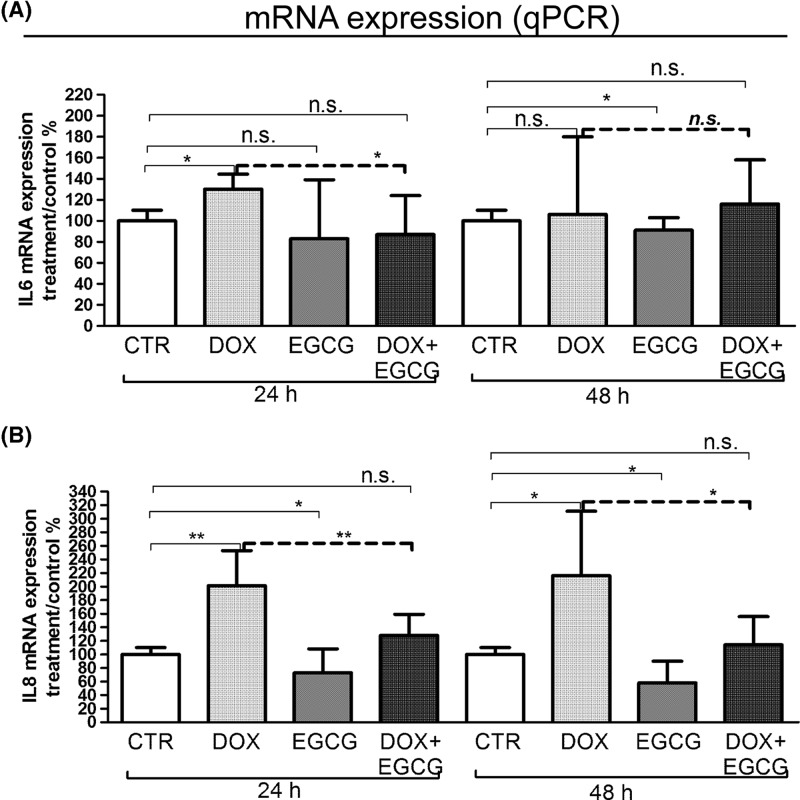
Effects of DOX and EGCG on interleukin mRNA expression in the ovarian tissue qRT-PCR for IL-6 and IL-8. RNA was isolated after 24 and 48 h treatment with DOX 1 μg/ml, EGCG 10 μg/ml, and combined treatment with DOX+EGCG at the aforementioned concentrations. IL-6 (**A**) and IL-8 (**B**) mRNA expression was shown as a percentage of treated on control samples (CTR). Each bar represents the mean (± S.D.) of three independent experiments. *P<0.05, **P<0.01, n.s.: not significant.

**Figure 3:**
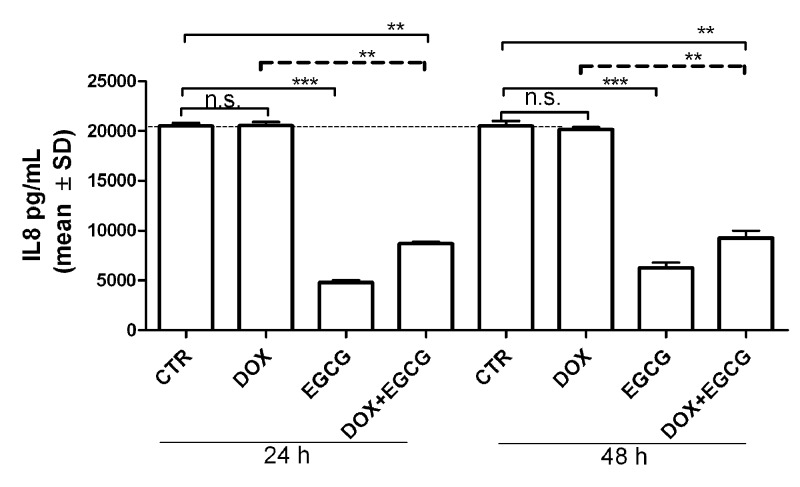
Effects of DOX and EGCG on IL-8 production by the ovarian tissue Multiplex bead-based immunoassay for IL-8 after 24 and 48 h treatment with DOX 1 μg/ml, EGCG 10 μg/ml, and combined treatment with DOX+EGCG at the aforementioned concentrations. IL-8 production was expressed as pg/ml. Each bar represents the mean (± S.D.) of three independent experiments. **P<0.01, ***P<0.001, n.s.: not significant.

IL-6 mRNA expression significantly increased after 24 h of treatment with DOX (*P*<0.05). EGCG alone slightly reduced (not significant, n.s.) the IL-6 mRNA expression after 24 h and significantly after 48 h of treatment (*P*<0.05). When administered with DOX, EGCG blocked the DOX-induced IL-6 mRNA expression after 24 h (*P*<0.05) but not after 48 h (Figure 2A). In parallel, DOX induced a significant increase of IL-8 mRNA expression after 24 h (*P*<0.01) and 48 h (*P*<0.05) of treatment. EGCG alone reduced significantly IL-8 expression when compared with CTR after both 24 and 48 h of treatment (*P*<0.05) and blocked the DOX-IL-8 induction after 24 h (*P*<0.01) and 48 h (*P*<0.05) (Figure 2B). By multiplex bead-based immunoassay, IL-6 and TNF-α were not detected in our experimental condition. IL-8 interleukin secretion after 24 and 48 h was slightly increased by DOX (n.s.) and significantly reduced by EGCG (*P*<0.001) with respect to CTR. Moreover, EGCG significantly reduced DOX-induced IL-8 secretion (*P*<0.05) (Figure 3). This result confirmed mRNA expression data.

### Effects of DOX and EGCG on metalloproteinase expression and activity

Inflammation is a trigger of several molecules involved in physiological processes such as metalloproteinases of matrix (MMPs). The expression of MMPs, in particular of gelatinases (MMP9 and MMP2), plays an important role in matrix remodeling during ovarian pathophysiological condition [44].

**Figure 4:**
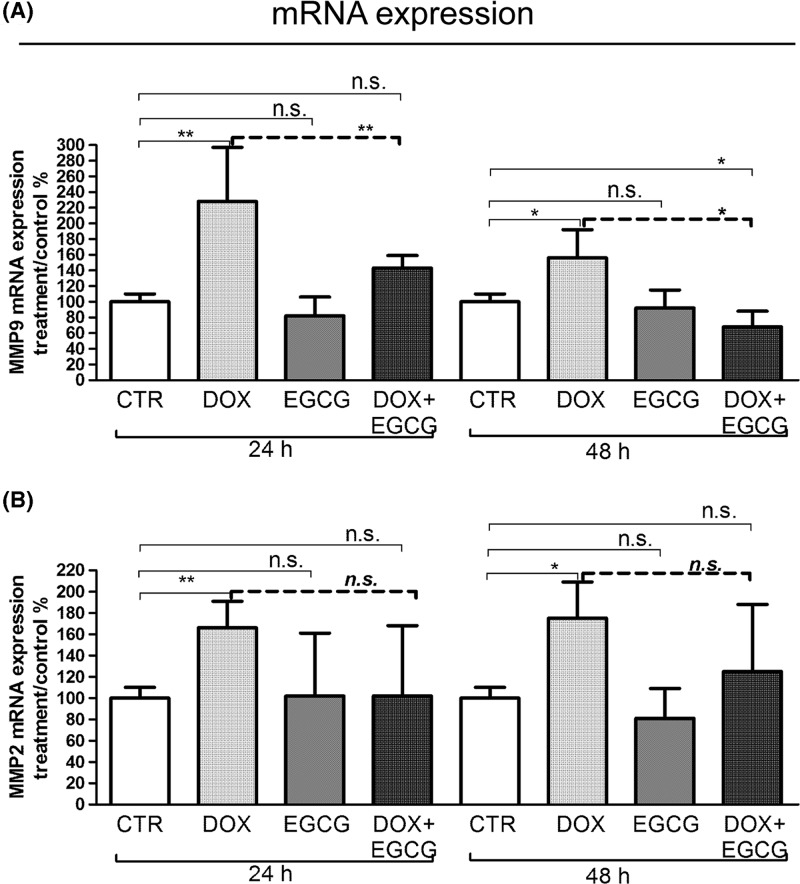
Effects of DOX and EGCG on MMP mRNA expression in the ovarian tissue qRT-PCR for MMP2 and MMP9. RNA was isolated after 24 and 48 h treatment with DOX 1 μg/ml, EGCG 10 μg/ml, and combined treatment with DOX+EGCG at the aforementioned concentrations. MMP9 (**A**) and MMP2 (**B**) mRNA expression was shown as a percentage of treated on control samples (CTR). Each bar represents the mean (± S.D.) of three independent experiments. *P<0.05, **P<0.01, n.s.: not significant.

Therefore, we tested the mRNA expression and activity of MMP9 and MMP2 after DOX treatment alone or in combination with EGCG. A significant increase of MMP9 and MMP2 mRNA expression was observed after 24 h (*P*<0.01) and 48 h (*P*<0.05) DOX treatment (Figure 4A,B). When EGCG was administered together with DOX, MMP9 (*P*<0.01 after 24 h and *P*<0.05 after 48 h) and MMP2 (n.s.) DOX-induced mRNA expression decreased (Figure 4A,B).

**Figure 5:**
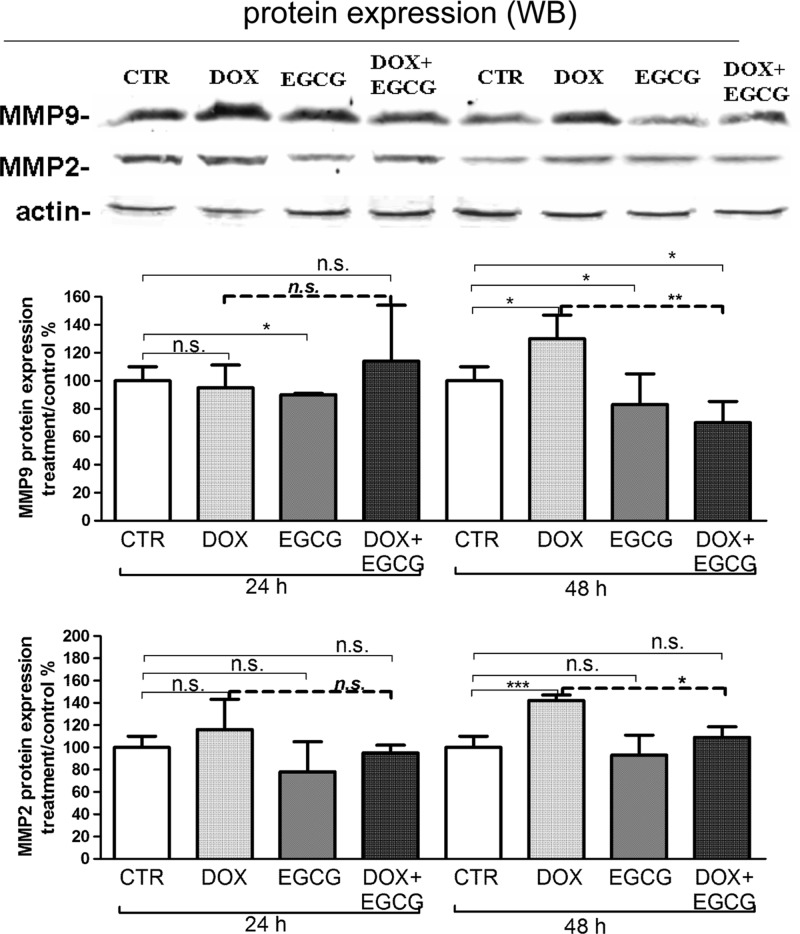
Effects of DOX and EGCG on MMP protein expression in the ovarian tissue Western blot for MMP2 and MMP9. Proteins were isolated after 24 and 48 h treatment with DOX 1 μg/ml, EGCG 10 μg/ml, and combined treatment with DOX+EGCG at the aforementioned concentrations. MMP9 (**A**) and MMP2 (**B**) protein expression was shown as a percentage of treated on control samples (CTR). Each bar represents the mean (± S.D.) of three independent experiments. *P<0.05, **P<0.01, ***P<0.001, n.s.: not significant.

Using western blot analysis, a significant increase of MMP9 (*P*<0.001) and MMP2 (*P*<0.05) protein expression was observed after 48 h of DOX treatment (Figure 5A,B). EGCG reduced the MMP9 expression significantly after 24 and 48 h of treatment when compared with CTR (*P*<0.05). When co-administered with DOX, EGCG reduced DOX-induced MMP9 (*P*<0.01) and MMP2 (*P*<0.05) expression after 48 h (Figure 5A,B).

**Figure 6:**
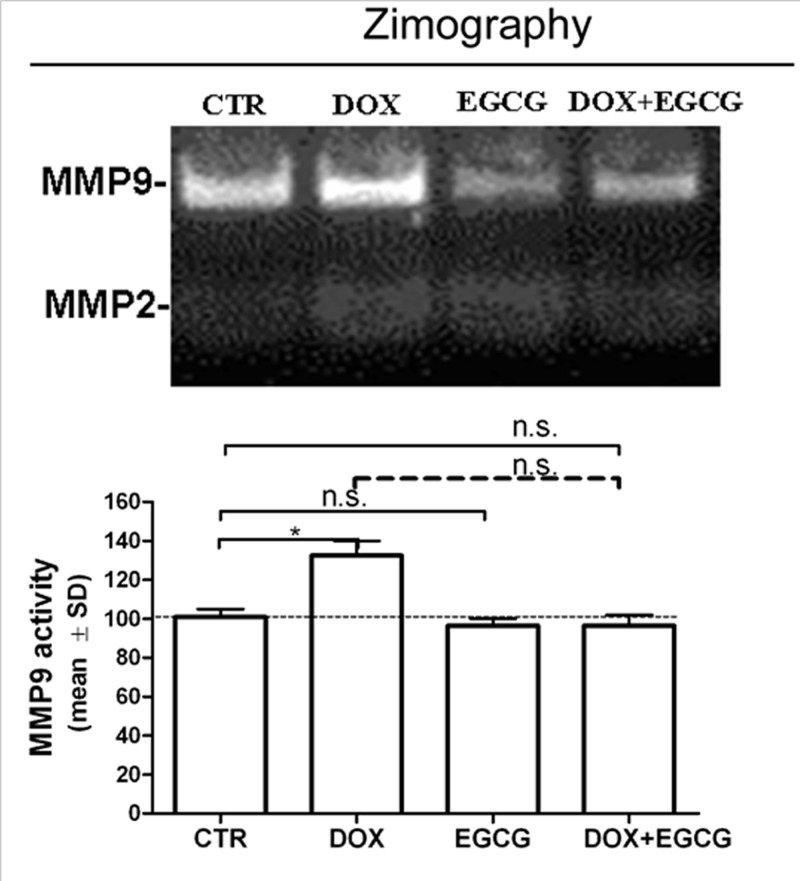
Effects of DOX and EGCG on MMP activity in the ovarian tissue Zymography for MMP9 activity. Tissue was treated with DOX 1 μg/ml, EGCG 10 μg/ml, and combined treatment with DOX+EGCG at the aforementioned concentrations for 24 h. Each bar represents the mean (± S.D.) of three independent experiments about MMP activity. *P<0.05, n.s.: not significant.

At last, we analyzed the effect of DOX on MMP9 and MMP2 activity using zimography. We confirmed a DOX-induced MMP9 activity (*P*<0.05) after 24 h. MMP9 activity remained similar to CTR when EGCG was administered alone or in association with DOX (n.s.). MMP2 activity was not detected in our experimental condition (Figure 6).

### Effects of DOX and EGCG on human ovarian tissue preservation and viability

A total of 185 follicles (50 from CTR, 43 from DOX, 47 from EGCG, and 45 from DOX+EGCG) was analyzed using light microscopy. The majority of follicles was primordial and intermediary (97.2 ± 2.5%), whereas the remaining were primary and secondary (2.8 ± 1.6%).

**Figure 7:**
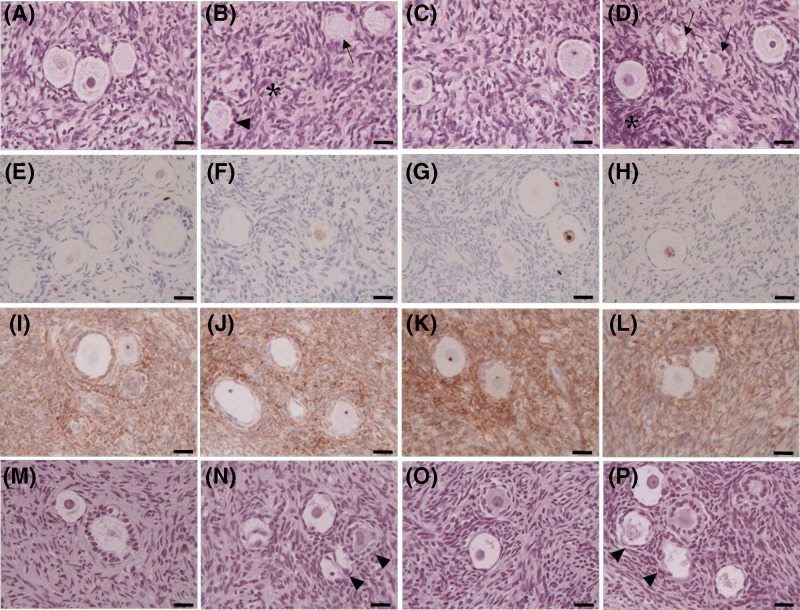
Effects of DOX and EGCG on preservation and viability of the ovarian tissue Light microscopy of ovarian tissue after 24 h treatment with DOX 1 μg/ml, EGCG 10 μg/ml, and combined treatment with DOX+EGCG at the aforementioned concentrations. (**A–D**) Hematoxylin/eosin staining of untreated tissue-CTR (A) and treated with DOX (B), with EGCG (C), with DOX+EGCG (D). (**E–H**) Immunohistochemical staining for ki-67 of CTR tissue (E) and tissue treated with DOX (F), with EGCG (G), with DOX+EGCG (H). (**I–L**) Immunohistochemical staining for Bcl2 of CTR tissue (I) and tissue treated with DOX (J), with EGCG (K), with DOX+EGCG (L). (**M–P**) TUNEL assay of untreated tissue-CTR (M) and treated with DOX (N), with EGCG (O), with DOX+EGCG (P). Irregular-shaped follicles (arrowheads); follicles with cytoplasmic vacuoles (arrows); areas with pyknotic nuclei of stromal cells (asterisks). Scale bar = 25 µm.

Regarding the tissue preservation, after 24 h of culture, CTR stromal cells showed an intact architecture: oval- to spindle-shaped nuclei, dispersed chromatin without interstitial oedema. Follicles showed regular shape and close adherence between oocyte and granulosa cells. The oocytes had mitochondria clustered around the nucleus, homogeneous cytoplasm, and no vacuoles were observed (Figure 7A). Tissue morphology was also maintained after 48 h of culture.

After 24 h of DOX supplementation, areas with pyknotic stromal cells were present. Follicles appeared with irregular shapes and showed oocytes with cytoplasmic vacuoles (Figure 7B). Similar characteristics were observed after 48 h of treatment.

The ovarian tissue cultured in presence of EGCG for 24 and 48 h showed stroma and follicles morphologically comparable with those observed in CTR samples (Figure 7C).

When co-administered with DOX, EGCG improved tissue morphology during the culture period: some areas with piknotic stromal cells persisted; some follicles had a well-preserved morphology with central round nucleus and regular cytoplasmic organelles while others showed irregular shapes and cytoplasmic vacuoles (Figure 7D). The mean percentages of damaged follicles in the different experimental groups after 24 h of culture were as follows: CTR 23.6 ± 7.4%; DOX 42.8 ± 5.4%: EGCG 25.9 ± 2.7%, DOX + EGCG 29.2 ± 4.5% (CTR vs DOX *P*<0.05; EGCG vs DOX *P*<0.05; DOX vs DOX+EGCG *P*<0.05; CTR vs EGCG n.s.; CTR vs DOX+EGCG; EGCG vs DOX+EGCG n.s.). After 48 h of culture, the percentages of damaged follicles were comparable with those observed after 24 h.

We also assessed the proliferative status and the viability of the ovarian cell population by ki-67 and Bcl2 immunostaining. A positive ki-67 nuclear staining was observed in most nuclei of oocytes and granulosa cells of growing follicles in all experimental conditions after both 24 and 48 h of culture. No positive staining was found in stromal cells (Figure 7E–H). Stromal tissue was diffusely Bcl2 stained after both 24 and 48 h of culture in all experimental groups. Positive staining for the antiapoptotic protein was observed in the granulosa cells of growing follicles (Figure 7I–L).

TUNEL assay was also carried out to evaluate a possible response of the ovarian tissue to chemotherapy drug treatment through apoptosis. No TUNEL-positivity was found in follicles (oocytes and granulosa cells) nor in the stromal cells of all experimental groups after 24 and 48 h of culture (Figure 7M–P).

**Figure 8:**
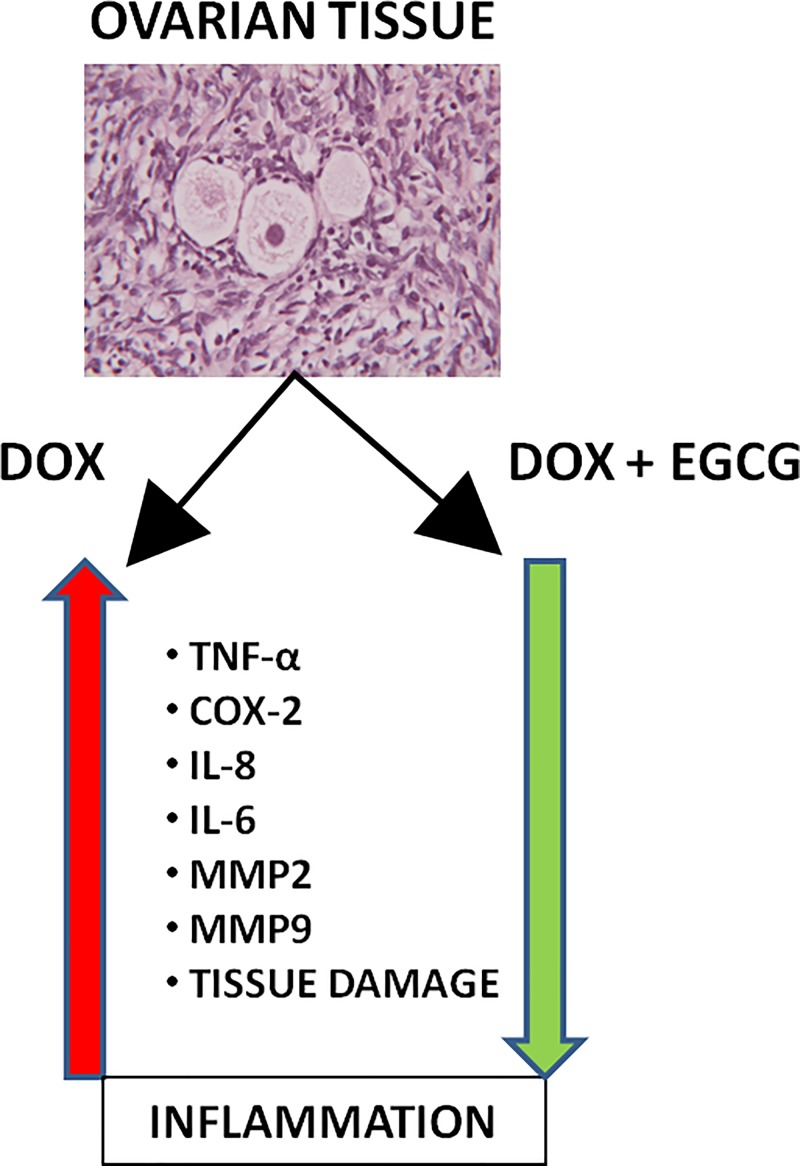
Effects of EGCG on DOX-induced inflammation on the ovarian tissue DOX induces the expression of pro-inflammatory cytokines, such as TNF-α and COX-2, and of ILs, such as IL-6 and IL-8 and of inflammatory proteins mediators MMP2 and MMP9. EGCG can block this pathway.

The effects of DOX and/or EGCG treatment on human ovarian tissue have been summarized in Figure 8.

Bcl2 protein expression was also analyzed by western blot. No statistical differences in Bcl2 expression after 24 and 48 h of DOX treatment were observed. Also EGCG alone did not affect Bcl2 expression compared with control. EGCG supplemented with DOX slightly reduced the protein levels compared with those of the DOX alone at 24 and 48 h of culture (n.s., Supplementary Figure S3). This data confirmed the histological observations. Bax protein expression was not detected in our experimental condition (data not shown). EGCG and DOX did not have effects on Poly PARP activation, the main protein involved in apoptosis activation (n.s., Supplementary Figure S3).

## Discussion

DOX is a potent chemotherapic drug and its gonadotoxicity can destroy the reproductive potential of young cancer patients, with physical and social implication on their quality of life after recovery. Therefore, the knowledge of the processes involved in DOX-induced ovarian toxicity and alternatives to reduce its side effect have a significant clinical relevance.

Since inflammation plays a key role in DOX toxicity, we investigated the inflammatory responses of the ovary to DOX treatment in the human model and the ability to reduce its toxicity using EGCG, the main catechin of the green tea.

Our results showed that DOX induced inflammation in the human ovarian tissue, as indicated by an increase in expression and activity of specific inflammatory cytokines and ILs, such as TNF-α, COX-2, IL-6, and IL-8. Furthermore, a significant increase in the matrix metalloproteinases MMP9 and MMP2 was found after DOX treatment. MMPs play a role in the pathogenesis of inflammatory diseases with focal tissue destruction [45–47]. Recent findings indicate that MMPs act on pro-inflammatory cytokines, chemokines, and other proteins to regulate various aspects of inflammation and immunity. In fact, one potential action of MMPs is to convert chemokines from their true (or initial) nature as chemotactic molecules into antagonistic derivatives, thereby disrupting the further recruitment of cellular components and contributors to sites of inflammation [47]. Therefore, stimulation of inflammatory cells by cytokines and ILs may induce the production of MMPs. In turn, the activation of MMPs may regulate the recruitment and the availability of inflammatory mediators [48]. In fact, it has been demonstrated that MMPs are involved, not only in the matrix degradation, but also in the regulation of growth factors [49], apoptotic factors [50], and cell-surface receptors [51].

In attempt to characterize the inflammation effect of DOX on the human ovarian components, we performed a histological ovarian tissue examination observing morphological changes induced by DOX in the stroma as well as in the follicles. In particular, areas with pyknotic stromal cells and follicles with irregular shapes and oocyte cytoplasmic vacuoles were observable. The percentage of damaged follicles was significantly higher after DOX exposure compared with CTR group.

Similar data are described in other *in vitro* studies showing microvascular alteration, stroma damages, and follicle loss [10,11]. Parenchimal alteration and cortical fibrosis were also reported by histological analysis of ovarian biopsies taken from women receiving chemotherapy [52]. In an our previous study, DOX induced growth inhibition in human primary ovarian stromal cells through the activation of apoptosis [38]. In the present study, DOX did not alter the proliferative status (ki-67 and Bcl2 expression) of tissue. The monoclonal ki-67 antibody recognizes a nuclear antigen that is expressed in all stages of the cell cycle except G_0_, so it represents a good index of the capacity of tissue to proliferate. Apoptosis-inhibiting Bcl2 is also associated with mitotic proliferation. A positive staining found in most follicles, suggests that cell proliferation is maintained. Furthermore, TUNEL analysis failed to find evidence of increased apoptosis in the ovarian cell population after DOX exposure.

We hypothesize that the ovarian damage can depend on the duration and dose of chemotherapeutic treatment. After 24–48 h of treatment with 1 µg/ml DOX, the drug induced the activation of tissue inflammatory responses; the DNA damage induced by DOX could be not sufficient to cause cell death or DNA repair mechanisms could prevent cell death.

Moreover, it is important to consider that the ovary is a heterogeneous organ having follicles at different developmental stages, ranging from quiescent primordial follicles to growing antral follicles. It is possible that these distinct follicle classes respond differently to DOX based on cell division rates and metabolic activity. Therefore, primordial follicles may respond to DOX via the oxidative stress pathway, while growing follicles may respond to DOX via apoptosis pathway [53].

Morgan et al. [54] found that DOX (0.1, 0.5, 1, and 5 µg/ml) preferentially caused damage to the granulosa cells in a mouse ovary culture system. Few primordial follicles were left after exposure to high levels of the drug, but TUNEL assay did not evidence increased apoptosis within primordial follicles [54]. Roti Roti and Salish [53] reported DNA damage in primary granulosa cells as well as granulosa and theca/stromal cells from cultured mouse ovaries after treatment with DOX (500, 50, and 10 µM). The same research group translated acute DOX ovarian insult from mouse to marmoset ovary demonstrating that DOX induced DNA damage and subsequent H2AX activation in granulosa cells of antral follicles and corpora lutei, while with low frequency in granulosa cells of preovulatory follicles (primordial, primary, and secondary) [55].

In our human ovary culture system, we investigated for the first time the EGCG for ovarian protection during DOX exposure. The potential protective role of green tea polyphenols, including EGCG, during DOX therapy, without side effects, was investigated by some studies in other organs, such as heart and small intestine [56,57]. Treatment with DOX significantly decreased cardiomyocyte viability and induced apoptosis after treatment. EGCG protected myocytes against oxidative stress-induced cellular injury in DOX-treated cardiac myocytes, in a concentration-dependent manner. EGCG also improved contractile function of DOX-treated cardiomyocytes: EGCG increased the amplitudes of both electrically- and caffeine-induced Ca2+ transients in DOX-treated myocytes, suggesting that EGCG may protect heart against DOX-induced myocyte injury by improving Ca2+ handling through scavenging reactive oxygen species [56].

According to another study [57], intraperitoneal treatment with the chemotherapeutic agent irinotecan (IT) induced oxidative stress and inflammation in the small intestinal mucosa, as demonstrated by ileum glutathione concentration drop and increase in macrophage inflammatory protein-2 content, myeloperoxidase activity, and nuclear factor-κB translocation. Pathological changes and signs of inflammation were also observable histologically. Green tea polyphenols supplied orally prevented the IT-induced increase of glutathione disulfide and myeloperoxidase activity in the ileum, whereas lipid peroxidation was unaffected.

In the present study the co-treatment with EGCG significantly reduced the inflammation caused by DOX in the human ovarian tissue, dampening DOX-induced cytokine, IL and MMP expression and activity. EGCG did not alter tissue morphology during the culture period and significantly reduced DOX-induced follicle damage. Based on these results, EGCG seems to maintain the integrity of the ovarian tissue when compared with the control and to improve the preservation of follicles when co-administrated with DOX. However, further experiments have to be conducted in order to confirm the protective and antioxidant effect of the EGCG on human ovaries as the future perspectives.

## Conclusion

For the first time in human ovary, the present study shows that DOX administration causes production of pro-inflammatory cytokines, inflammatory ILs, and metalloproteinases that could be responsible for the ovarian function impairment of chemotherapy. The results of the study also provide evidence for the potential protective role of EGCG in reducing DOX-mediated inflammatory responses in the human ovarian tissue. Therefore, EGCG may be a promising ferti-protective agent against DOX gonadotoxicity in human cancer patients.

## Supporting information

**Supplementary Figure S1:** 

**Supplementary Figure S2:** 

**Supplementary Figure S3:** 

**Supplementary Table 1:** Primer sets used for RT-PCR analysis.

**Supplementary Table 2:** List of antibodies used in Western Blot analysis.

## Abbreviations

BCM: basal culture medium
BSA: bovine serum albumine
COX-2: cyclooxigenase-2
CTR: control group
DOX: doxorubicin
EGCG: epigallocatechin-3-gallate
IL: interleukin
IT: irinotecan
MMP: metalloproteinase
PBS: phosphate buffered saline
POF: premature ovarian failure
qRT-PCR: quantitative real-time PCR
TBS: transfer buffer saline
TNF: tumor necrosis factor
