# Supplementary material for: Epigallocatechin-3-gallate inhibits doxorubicin-induced inflammation on human ovarian tissue
Source: Biosci Rep. 2019 May 14;39(5):BSR20181424. doi: 10.1042/BSR20181424 (PMC6522724; doi:10.1042/BSR20181424)
Supplement: Supplementary file 1 [file BSR-2018-1424_suppS1.pdf]

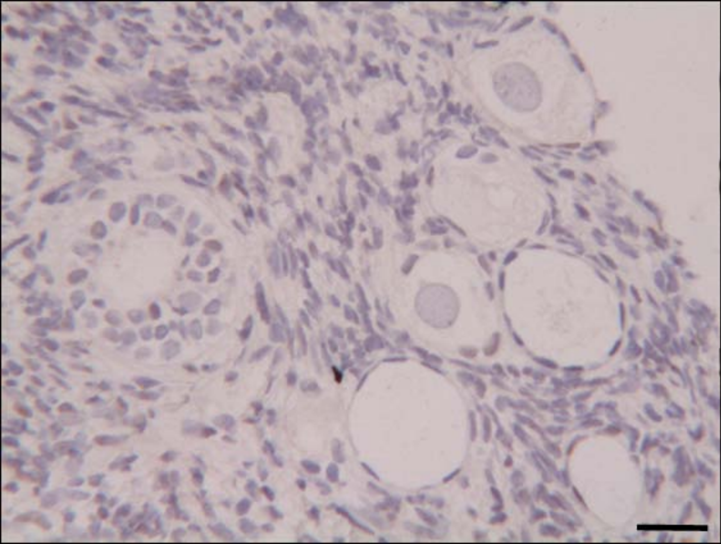

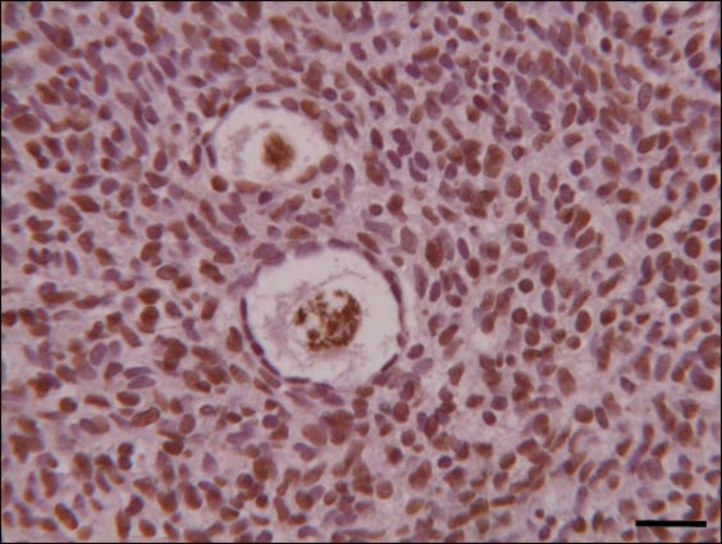

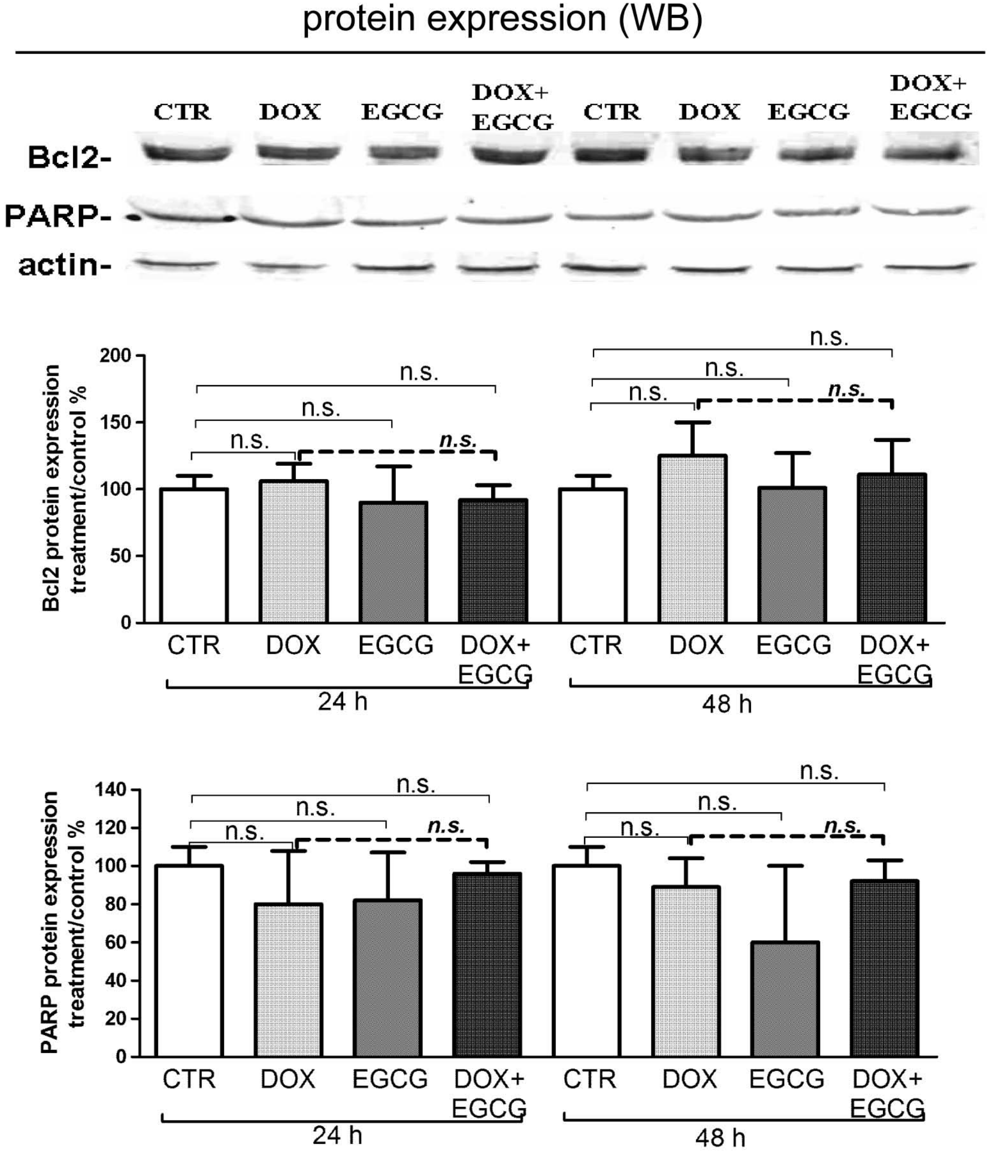
protein expression (WB)
CTR
DOX
EGCG
DOX+ EGCG
CTR
DOX
EGCG
DOX+ EGCG
Bcl2-
PARP-
actin-
Bcl2 protein expression treatment/control %
200
150
100
50
0
n.s.
n.s.
n.s.
n.s.
n.s.
n.s.
n.s.
n.s.
CTR
DOX
EGCG
DOX+ EGCG
CTR
DOX
EGCG
DOX+ EGCG
24 h
48 h
PARP protein expression treatment/control %
140
120
100
80
60
40
20
0
n.s.
n.s.
n.s.
n.s.
n.s.
n.s.
n.s.
n.s.
CTR
DOX
EGCG
DOX+ EGCG
CTR
DOX
EGCG
DOX+ EGCG
24 h
48 h

Supplementary Table 1. Primer sets used for RT-PCR analysis.

Abbreviations: MMP2: metalloproteinase 2; MMP9: metalloproteinase 9; COX-2: cyclooxygenase-2; TNF-α: Tumor necrosis factor alpha; IL-6: Interleukin 6; IL-8: Interleukin 8.

Primer	T(°C) annealling	Bp amplification	Forward 5'-3'	Reverse 5'-3'
**actin**	60	192	GGCATCGTGATGGACTCCG	GCTGGAAGGTGGACAGCGA
**MMP2**	63	139	ATCACATACAGGATCATTGGCTAC	TGATGTCTGCCTCTCCATCA
**MMP9**	63	180	ACGCCGCTCACCTTCACTC	GGACCACAACTCGTCATCGTC
**COX-2**	60	162	CCTGTGCCTGATGATTGC	CTGATGCGTGAAGTGCTG
**TNF-α**	60	110	GGCCCAGGCAGTCAGATCAT	GGGGCTCTTGATGGCAGAGA
**IL-6**	60	360	GAGAAAGGAGACATGTAACAAGAGT	GCGCAGAATGAGATGAGTTGT
**IL-8**	60	220	GCTTTCTGATGGAAGAGAGC	GGCACAGTGGAACAAGGACT

Supplementary Table 2. List of antibodies used in Western Blot analysis.

Antibody	Dilution	Type	Manufacturer
**anti-Bcl2**	1:1000	monoclonal mouse	Trevigen, USA
**anti-Bax**	1:1000	monoclonal mouse	Trevigen, USA
**anti-PARP**	1:1000	monoclonal mouse	Thermo Scientific, USA
**anti-MMP2**	1:500	polyclonal rabbit	S. Cruz, USA
**anti-MMP9**	1:500	polyclonal rabbit	S. Cruz, USA
**anti-actin**	1:1000	polyclonal rabbit	Sigma, USA
**Cy3**	1:2500	secondary conjugated	GE, Healthcare, UK
**Cy5**	1:2500	secondary conjugated	GE, Healthcare, UK

Abbreviations: Bcl2: B-cell lymphoma 2; Bax: Bcl-2-associated X; PARP: Poli ADP-ribosio polimerasi; MMP2: metalloproteinase 2; MMP9: metalloproteinase 9; Cy3: cyanine dye 3 conjugate; Cy5: cyanine dye 3 conjugate.
